# Cultural adaptation and feasibility of action over inertia in Japan: a multi-site pilot intervention study

**DOI:** 10.3389/fpsyt.2026.1851780

**Published:** 2026-06-11

**Authors:** Aiko Hoshino, Yasuhisa Nakamura, Takafumi Morimoto, Sachie Tanaka, Hikari Isaji, Ichiro Kutsuna, Kojiro Kawano

**Affiliations:** 1 Graduate School of Medicine, Nagoya University, Nagoya, Japan; 2 Department of Rehabilitation, Faculty of Health Sciences, Nihon Fukushi University, Handa, Japan; 3 Department of Occupational therapy, School of Health Sciences, Sapporo Medical University, Sapporo, Japan; 4 Department of Occupational Therapy, Shinshu University of Health Sciences, Matsumoto, Japan; 5 Department of Occupational Therapy, Ibaraki Prefectural University of Health Sciences, Ibaraki, Japan; 6 Occupational Therapy Course, Faculty of Rehabilitation and Care, Seijoh University, Tokai, Japan; 7 Department of Occupational Therapy, Saitama Prefectural University, Saitama, Japan

**Keywords:** occupational therapy, recovery, schizophrenia, serious mental conditions, time-use

## Abstract

**Introduction:**

Action Over Inertia (AOI) is a recovery-oriented intervention designed to promote occupational engagement and support personal recovery through changes in everyday activity patterns. While AOI has been examined in Western contexts, its applicability and clinical adaptation in East Asian settings remain underexplored. This study used a mixed-methods approach to examine the feasibility and cultural adaptation of a group-based AOI program in Japan.

**Methods:**

A mixed-methods pilot feasibility design was used. The quantitative component was a single-arm repeated-measures pilot study conducted across four psychiatric daycare facilities in Japan. Outcomes were assessed at baseline, post-intervention, and 1-month follow-up in 12 participants with serious mental illness using the Recovery Assessment Scale (RAS), Temple University Community Participation Measure-Japanese version (TUCP-J), and Social Functioning Scale (SFS), alongside symptom severity assessed with the Brief Psychiatric Rating Scale (BPRS). These measures were used as exploratory clinical outcomes to describe preliminary patterns of change and to inform future controlled studies, rather than to determine intervention effectiveness. The qualitative component involved semi-structured interviews with occupational therapists who delivered the intervention, to contextualize the quantitative findings and identify implementation barriers and adaptations. The quantitative intervention study was approved by the Research Ethics Committee of the Graduate School of Medicine, Nagoya University, Japan (2023-0209), and registered with the UMIN Clinical Trials Registry (UMIN000045392). The qualitative interview study was approved by the Ethics Review Committee for Research Involving Human Participants, Nihon Fukushi University, Japan (23-041-03).

**Results:**

No significant improvements were observed in the exploratory outcomes of recovery, participation, or social functioning. Although BPRS showed a significant overall time effect, post-hoc comparisons between time points were not significant, and this change cannot be causally attributed to AOI because of the single-arm design. In an exploratory observation, participants with higher symptom severity appeared to show lower community participation. Qualitative findings suggested that the intervention dose, participant–program fit, readiness for activity change, and group-based delivery conditions may need further optimization within Japanese psychiatric daycare settings.

**Discussion:**

The findings suggest that group-based AOI may be feasible to implement in Japanese psychiatric daycare settings, but further cultural and clinical adaptation is needed before effectiveness can be evaluated in controlled trials. Adaptation may require preserving AOI’s theoretical foundations while making the intervention less exposing, less abstract, and more accessible in group-based settings. Future studies should use controlled designs, longer follow-up periods, and broader outcome indicators to examine the effectiveness and implementation of culturally adapted AOI.

**Clinical trial registration:**

UMIN Clinical Trials Registry https://www.umin.ac.jp/ctr/, identifier UMIN000045392.

## Introduction

Recovery for people with severe mental illness (SMI) is understood as comprising multiple dimensions, including clinical recovery, social recovery, and personal recovery (1). Of them, clinical recovery, which aims to manage symptoms and improve cognitive functioning, has accumulated a certain level of evidence, particularly through interventions such as cognitive remediation (2). Similarly, in relation to social recovery, especially in the domain of employment support, empirically supported intervention models such as Individual Placement and Support (IPS) (3) and Illness Management and Recovery (IMR), which incorporates psychoeducation and peer support, have increasingly been established (4).

Personal recovery, by contrast, refers not only to symptom reduction or functional improvement, but to a deeply personal and unique process through which individuals change their attitudes, values, feelings, goals, skills, and roles and develop a way of living a satisfying, hopeful, and contributing life, even with the limitations caused by illness (5). A systematic review and narrative synthesis by Leamy et al. (1) further conceptualized personal recovery as comprising five key processes: connectedness, hope and optimism about the future, identity, meaning in life, and empowerment, known as the CHIME framework. The same review also noted that recovery may be shaped by cultural contexts, including culturally specific facilitating factors and collectivist notions of recovery. In the Japanese context, Kanehara et al. (6) showed that personal recovery among mental health service users is closely shaped by relationships with others, places where individuals can feel accepted and connected, and experiences of stigma. In addition, van Weeghel et al. (7) emphasized that personal recovery is complementary to, but distinct from, clinical recovery and represents a process rather than merely an outcome. Together, these studies suggest the need for intervention approaches that move beyond symptom reduction and functional improvement to support recovery through everyday life, relationships, meaning, and self-directed participation.

Despite this conceptual development, support techniques and intervention models that focus on personal recovery, namely, recovery achieved through changes in the person’s everyday life itself, remain less well developed in terms of both structured methodology and empirical evidence. Interventions that attend to daily activities and patterns of time use, and that seek to promote recovery through transformation of everyday life, are still limited as systematically developed models, despite growing recognition of their theoretical importance.

Action Over Inertia (AOI) was developed as one theoretical and practical response to this gap, and a second edition of the manual has now been published (8). AOI aims to promote health and well-being by reconnecting individuals who experience occupational imbalance and limited engagement in activity with meaningful occupations. Previous studies, conducted primarily in Australia and Canada, have examined its effectiveness (9, 10). However, these studies have been limited largely to Western contexts, and AOI has not yet been introduced or empirically examined in Asian countries, including Japan.

In Japan as well, practices related to clinical recovery, such as cognitive rehabilitation, and those oriented toward social recovery, such as IPS, have gradually become more widespread (11). However, intervention models that place personal recovery at the center and support changes in everyday life and the reconstruction of a self-directed daily life have rarely been structurally examined or empirically tested. Therefore, introducing and evaluating an intervention such as AOI in Japan is important for advancing the methodology of personal recovery-oriented support.

At the same time, introducing a recovery-oriented intervention model such as AOI, originally developed in Western countries, into Japan requires consideration not only of the fact that it has not yet been implemented there, but also of the medical, service, and cultural context of Japan itself. This is because Western countries and Japan differ substantially in their mental healthcare systems, the organization of support services, the forms of intervention practice, and the cultural values that shape helping relationships.

First, Japan has a healthcare system characterized by high insurance coverage and strong institutional support, allowing long-term medical support to be sustained (12). Though this system provides a relatively stable basis for care, it has also had the effect of constraining the development of recovery-oriented support grounded in community living and service users’ self-determined life choices. Indeed, the average length of psychiatric hospitalization in Japan (255.0 days) remains markedly longer than in Western countries such as Canada and Australia, even though community transition has progressed in recent years (13). Such a service structure may have privileged approaches aimed at maintaining stability within medical and welfare systems, rather than supporting recovery through experimentation and change in everyday life.

In addition, Japanese psychiatric rehabilitation has distinctive characteristics in the form of intervention practice itself. Owing to its historical development, many interventions in Japan, including occupational therapy, continue to be delivered primarily in group-based formats, whereas individual interventions are often limited to settings such as outreach or home-visit support. In other words, Japanese clinical practice tends to be organized around structured group programs rather than interventions centered on individualized reflection and goal setting. This practice structure poses an important consideration for AOI, which places the reconstruction of everyday life at its core.

Furthermore, beyond these institutional and structural differences, cultural factors also play an important role in intervention adaptation. Differences between the individualistic values often emphasized in Western societies and the collectivist values more commonly associated with Asian contexts, including Japan, are reflected in styles of self-expression, the formation of priorities, helping relationships, and patterns of communication (14). In recovery-oriented models, which emphasize articulating one’s values, hopes, priorities and collaboratively building support around them, such cultural differences are likely to fundamentally shape how an intervention is received and whether it can be implemented effectively. Cultural considerations are therefore particularly important when introducing recovery models centered on service users’ own life construction.

Previous research suggests that the meanings and functions of self-expression and self-disclosure differ between East Asian and Western contexts. In East Asian societies, the self is often understood as relationally constituted and defined through connections with others. Accordingly, self-expression may be less likely to be framed as an individual right, as it often is in Western societies such as the United States, and more likely to be embedded in social roles and relationships. This cultural background may make the explicit expression of one’s internal self-less salient or less strongly valued, both by the individual and by others (15). Consistent with this view, studies have suggested that self-disclosure is generally less common in East Asian contexts than in Western ones (16). Because AOI includes multiple sessions that ask participants to articulate their hopes and their own ways of living, such cultural differences may be highly relevant to its implementation. From a cultural psychological perspective, expressing one’s hopes has been associated with increased commitment in Western contexts, whereas this effect may not emerge in the same way in East Asian contexts (15). Taken together, these findings suggest that cultural differences may influence not only how AOI is implemented, but also its effects.

To date, rehabilitation models developed in Western countries have often been introduced into Asian contexts primarily through translation. However, in interventions such as recovery-oriented support, which are deeply connected to everyday life, relationships, values, and role expectations, language translation alone is insufficient. Rather, implementation requires a process of cultural adaptation that encompasses multiple levels, including intervention content, delivery methods, communication styles, modes of participation, and ways of supporting goal setting.

Against this background, the present study introduced AOI, an intervention grounded in personal recovery, into Japan and conducted a multi-site pilot study in which it was delivered in a group-based format. The study aimed to evaluate the feasibility of group-based AOI in Japan and to examine the process of cultural adaptation, particularly the implementation barriers and adaptation challenges identified from the perspectives of occupational therapists. By exploring the lived experiences of implementing therapists, it also sought to contextualize the quantitative findings in relation to the cultural and clinical adaptability of the intervention.

## 2 Materials and methods

This study employed a mixed-methods approach to examine the feasibility and cultural adaptation of a multi-site pilot implementation of AOI in Japan. The quantitative component was exploratory and was used to describe changes in recovery, social functioning, and participation over time, rather than to determine intervention effectiveness. The qualitative component was designed to contextualize these quantitative findings by exploring therapists’ experiences of implementation, with particular attention to cultural and institutional adaptation and implementation barriers. The integration of quantitative and qualitative findings was therefore conducted at the interpretation stage, with the qualitative findings used to contextualize exploratory quantitative trends and to identify implementation conditions relevant to future adaptation and trial design.

### 2.1 Quantitative component

A multicenter single-arm intervention study was conducted to examine the feasibility of delivering AOI in a group-based format across multiple facilities in Japan. Outcomes were assessed at baseline, post-intervention, and follow-up within a single cohort. Quantitative measures included recovery, assessed using the Recovery Assessment Scale (RAS), as well as measures of social functioning and participation. These measures were used to explore preliminary patterns of change and to inform the design of future controlled studies, rather than to provide definitive evidence of effectiveness.

### 2.2 Qualitative component

To explore the feasibility, cultural adaptability, and clinical applicability of AOI within the Japanese healthcare and rehabilitation context, semi-structured interviews were conducted with occupational therapists who played a central role in implementing the intervention. The interviews explored the perceived competencies required for implementation, as well as barriers and facilitators related to delivering and adapting AOI in Japanese psychiatric daycare settings.

### 2.3 Setting

The quantitative intervention study was conducted in daycare centers affiliated with four psychiatric hospitals in Japan. These centers are a common form of community psychiatric rehabilitation service in Japan, functioning both as a step toward community participation following hospital discharge and, in some cases, as a longer-term place of support and belonging. The centers operate five days per week, with morning and afternoon sessions, and provide approximately seven hours of rehabilitation programming per day. Most services are delivered in a group format. Typical programs include daily activities such as cooking and shopping, sports, psychoeducation, social skills training (SST), and leisure-based activities such as crafts. Based on the STAX-SA classification, these centers can be characterized as Type 1 settings, where programs are professionally led, participation occurs within a predefined service structure, and the use of information and communication technology (ICT) and assistive technologies is limited. Since these services are embedded within the Japanese medical system, opportunities for self-evaluation and self-determination may be relatively constrained. Previous studies have implemented AOI in Canadian assertive community treatment services for community-dwelling people with serious mental illness and in Australian community residential mental health rehabilitation services, including Community Care Units (9, 10). Compared with these AOI implementation contexts, Japanese psychiatric daycare services are more closely embedded within hospital-based medical care and organized around structured, time-tabled group programs. In this context, participation often involves attending the daycare for a fixed period of the day and following established rules and routines, including regular medical consultation and medication-related support. These features may provide continuity, safety, and a sense of belonging, but they may also foster relatively passive forms of participation and make it more challenging to introduce interventions that require individualized reflection, self-evaluation, and self-directed activity change.

Psychiatric daycare services in Japan are delivered by multidisciplinary teams, including psychiatrists, nurses, occupational therapists, psychologists, and psychiatric social workers. In the participating daycare centers, occupational therapists were core staff members because they were responsible for organizing and facilitating many of the daily group-based activity programs and for shaping the therapeutic structure of the daycare environment. While nurses were primarily involved in monitoring physical and mental health conditions and supporting medication-related needs, and psychiatric social workers provided support related to welfare services, financial issues, and access to community resources, occupational therapists played a central role in managing the activity-based programs through which participants spent much of their time in the daycare setting. The central involvement of occupational therapists in daycare programming should be distinguished from the nature and depth of the intervention typically provided. In routine practice, occupational therapists were extensively involved in planning and facilitating group-based programs and maintaining a safe and structured therapeutic environment. At the same time, such involvement did not necessarily include systematic support for individualized reflection on time use, exploration of personally meaningful activities, or development of self-directed activity changes beyond the daycare setting. Thus, AOI represented not simply an additional activity program, but a shift toward a more individualized, reflective, and recovery-oriented use of activity within an otherwise structured group-based service context.

Furthermore, according to a national survey conducted by the Ministry of Health, Labor and Welfare, 65.2% of psychiatric daycare users had used these services for at least one year (17), suggesting that these services may be oriented more toward maintaining stability within daycare attendance than toward promoting recovery-oriented community participation.

The qualitative interview study targeted occupational therapists employed in these daycare centers who had been centrally involved in the implementation of the intervention.

### 2.4 Participants

#### 2.4.1 Quantitative component

Participants were eligible if they met the following inclusion criteria: (a) were aged between 20 and 65 years; (b) had been diagnosed with a serious mental illness (SMI) according to the Diagnostic and Statistical Manual of Mental Disorders, Fifth Edition (DSM-5); (c) had attended a psychiatric daycare program affiliated with a medical institution for more than 1 year; (d) were attending daycare at least twice a week at the time of recruitment and were able to participate in group activities; and (e) had obtained permission from their treating psychiatrist to participate. Exclusion criteria were difficulty understanding the study explanation provided in both written and verbal forms, inability to provide informed consent, and comorbid diagnoses of substance dependence, intellectual disability, or organic brain disorder. Individuals with comorbid substance dependence were excluded because, in the Japanese psychiatric rehabilitation context, substance dependence is often addressed within a different treatment framework from that used for general psychiatric daycare services for people with SMI. In addition, substance dependence may involve specific clinical needs, including addiction-focused treatment and possible cognitive or organic difficulties, which could affect participants’ ability to understand and engage with the AOI program.

Participants were recruited through posters displayed at each facility and through invitations from staff members. Participation in the program itself was permitted regardless of study participation. This was done to respect the voluntary nature of research participation and avoid restricting access to the AOI program in routine clinical settings. Therefore, some AOI groups included both research participants and non-participants.

#### 2.4.2 Qualitative component

Participants in the qualitative interview study were occupational therapists who had played a central role in the intervention study. Only those who provided consent to participate were included.

### 2.5 Procedure

The program comprised 24 sessions over 3 months, delivered twice weekly, including 22 group sessions and two individual sessions at the beginning and end of the intervention. The individual sessions included interview-based reflection as well as the pre- and post-intervention assessments. In addition, semi-structured interviews with the occupational therapists who delivered the intervention were conducted within approximately 1 to 2 months after completion of the intervention. Details of each component are described below.

#### 2.5.1 Quantitative intervention study: group-based action over inertia

Participants received a 3-month group-based AOI intervention. AOI is an intervention specifically developed for people with SMI and is grounded in the recovery framework and the Do-Live-Well framework (18). The aim of the intervention is to connect individuals experiencing pervasive and persistent occupational imbalance and disengagement from occupation with meaningful activities to promote health and well-being. The intervention is delivered in a workbook-based format, in which sessions are repeated in relation to individualized goals.

As noted above, psychiatric daycare centers in Japan primarily provide group-based programs. Given that this format is also the most feasible considering staffing structures and reimbursement systems, the present study adopted a group-based format for the intervention. The second edition of AOI proposes not only an individual format, but also a group session format; in this study, the group session format was used as a reference. Group size was approximately 3 to 5 participants. Sessions were conducted in groups that included both individuals who had consented to participate in the study and cooperate in data collection, and those who participated only in the program. This group size was based on prior AOI implementation (10) in small groups and on the need to provide a person-centered intervention in which facilitators could attend to each participant’s activity patterns, reflections, and goals.

All sessions were conducted in Japanese using a translated Japanese version of the AOI workbook. The translation of the AOI workbook was undertaken by four occupational therapists: the first author, who had completed facilitator training provided by the developers; one occupational therapist with long-term residency experience in an English-speaking country; and two occupational therapists with clinical experience. During the translation process, cross-checking was conducted to examine the appropriateness of wording and conceptual consistency. Any questions or ambiguities that arose during translation were directly discussed with the AOI developers to ensure consistency with the authors’ original intentions and theoretical background.

The program was structured broadly around the following five components and consisted of 24 sessions delivered twice weekly over a 3-month period. The first and final sessions were conducted individually and included interviews as well as pre- and post-intervention assessments.

1. Reflecting with participants on their current activity patterns to enhance awareness of change and motivation
2. Experiencing small, immediately achievable changes and reflecting on them to examine the meaning of change and its relationship to health
3. Providing psychoeducation regarding the relationships of health, illness, and activity
4. Planning for longer-term changes and working toward these changes collaboratively
5. Reviewing longer-term changes and providing follow-up support for their maintenance

In the present study, with permission from the developers, Section 3 of the intervention (information provision) was adapted by incorporating psychoeducational sessions from the Illness Management and Recovery (IMR) program in addition to the translated Japanese AOI text. During the workbook translation process, many concerns were raised that Part 3 of the AOI workbook was relatively abstract and had limited comprehensibility and practical fit for Japanese service users and practitioners. Therefore, this study adopted selected IMR sessions as an alternative format, since these sessions addressed comparable themes while being more familiar within Japanese mental health practice. Specifically, the following part of Japanese psychoeducation session(s) based on Illness Management and Recovery (IMR), developed by the Substance Abuse and Mental Health Services Administration (SAMHSA) of the U.S. Department of Health and Human Services (HHS) were used: the modules addressing stress coping, knowledge about medication, and relapse prevention (19). The IMR materials were not used as a separate symptom-management intervention, nor was the program intended to be an AOI–IMR hybrid intervention. Rather, selected IMR materials were used only within the information provision component of AOI, as culturally and clinically familiar psychoeducational resources to support discussion of the relationships among illness, health, everyday activity, and participation. Throughout these sessions, facilitators were instructed to maintain the focus on participants’ activity patterns, engagement in meaningful activities, and participation in daily life, consistent with the core intent of AOI. The research team confirmed that the incorporated IMR session(s) were consistent with the theoretical intent and aims of the original AOI Section 3, particularly in addressing the relationship between activity and health, as well as the everyday difficulties and functional challenges associated with illness. Although the AOI workbook does not include a formal fidelity framework or checklist, the research team considered fidelity in relation to the core principles of AOI: supporting reflection on activity patterns, promoting meaningful activity engagement, and linking activity change with health and participation. Accordingly, the intervention evaluated in this study should be understood as a culturally adapted group-based AOI program rather than a strict fidelity-tested delivery of the original AOI manual.

Before implementation, the occupational therapists who delivered the intervention received an online overview of the entire program. They were then asked to study each session using researcher-developed, audio-recorded, instructional videos of approximately 15 minutes per session. Opportunities to ask questions were subsequently provided by the research team.

#### 2.5.2 Qualitative interview: semi-structured interviews with occupational therapists

Semi-structured online interviews of approximately 45 minutes were conducted with the occupational therapists who had taken the lead in delivering the intervention. The interviews explored the skills and clinical judgments they used as occupational therapists when implementing AOI, their ways of engaging with participants, strategies for program management, and barriers encountered during implementation. All interviews were audio-recorded.

### 2.6 Data collection

#### 2.6.1 Data collection for the quantitative component

Assessments were conducted at three time points: pre-intervention, post-intervention, and follow-up (1 month after the post-intervention assessment). The following were collected. As this was a pilot feasibility study rather than a definitive effectiveness trial, the quantitative measures were used as exploratory clinical outcomes to examine possible areas of change and to inform outcome selection for a future RCT. Recovery, assessed using the Recovery Assessment Scale (RAS), was treated as the key exploratory outcome because AOI is grounded in a personal recovery-oriented approach. Community participation, assessed using the Japanese Temple University Community Participation Measure (TUCP-J), was included as an important exploratory outcome because AOI aims to support meaningful activity engagement and participation in everyday and community contexts. Social functioning and symptom severity were also assessed to provide additional information on participants’ clinical and functional changes.

Baseline characteristics (pre-intervention only): Baseline demographic and clinical data were collected, including age, sex, primary diagnosis, living situation, medication dosage, years of education, primary source of income, cumulative days of daycare attendance, and cumulative days of hospitalization.

Recovery: Recovery was assessed using the Recovery Assessment Scale (RAS). The RAS is one of the most widely used self-report measures developed to assess personal recovery of people with mental illness. A Japanese version with established reliability and validity was used (20). The RAS was selected as the key exploratory outcome because AOI is grounded in a personal recovery-oriented approach and aims to support participants in reflecting on and changing their everyday activity patterns in personally meaningful ways.

Participation: Participation was assessed using the Japanese Temple University Community Participation Measure (TUCP-J). The TUCP-J is a self-report measure of community participation by people with SMI, and the Japanese version has demonstrated good test-retest reliability (21). The TUCP-J was included as an important exploratory outcome because AOI aims to promote meaningful activity engagement and participation in everyday and community contexts.

Social functioning: Social functioning was assessed using the Social Functioning Scale (SFS), which was developed to comprehensively evaluate social functioning, and the Global Assessment of Functioning (GAF), an observer-rated measure. The GAF was rated by the treating psychiatrist. Japanese versions with established reliability and validity were used for both measures (22, 23). These measures were included as additional exploratory outcomes to capture broader functional changes that might be associated with changes in activity engagement.

Symptoms: Symptom severity was assessed using the Brief Psychiatric Rating Scale (BPRS). A Japanese version with established reliability and validity was used (24). The BPRS was included as an additional exploratory outcome to describe changes in psychiatric symptoms during the intervention period.

#### 2.6.2 Data collection for the qualitative component

Individual interviews were conducted with occupational therapists who had played a central role in implementing the intervention. Data were collected through semi-structured online interviews.

### 2.7 Analyses

#### 2.7.1 Quantitative analysis

For the intervention study, scores for all outcome measures were compared across three time points: baseline, post-intervention, and follow-up. Friedman tests were primarily used to examine differences in scores across the three assessments. When a significant difference was identified, *post hoc* pairwise comparisons were conducted.

#### 2.7.2 Qualitative analysis of interviews with occupational therapists

Interview data were transcribed verbatim and analyzed using content analysis. First, descriptions related to occupational therapists’ practices, perceptions, and difficulties in implementing AOI were extracted as the smallest meaningful units and coded. These codes were then compared in terms of similarities and differences in meaning and organized into subcategories and categories. Finally, the findings were organized into two overarching themes: competencies used in AOI delivery, and contextual and cultural adaptations and implementation barriers in Japanese psychiatric daycare settings. Within each theme, categories and subcategories were developed to capture similarities and differences in meaning.

### 2.8 Ethics approval

Ethical approval for the quantitative intervention study was obtained in 2023 from the Research Ethics Committee of the Graduate School of Medicine, Nagoya University, Japan (authorization number: 2023-0209). The study was also registered with the UMIN Clinical Trials Registry (UMIN-CTR) (UMIN study ID: UMIN000045392).

Ethical approval for the qualitative interview study was obtained from the Ethics Review Committee for Research Involving Human Participants, Nihon Fukushi University (approval number: 23-041-03). All participants provided written, informed consent after receiving both written and verbal explanations of the study.

## 3 Results

### 3.1 Quantitative component

#### 3.1.1 Participant flow and baseline information

**Figure 1:**
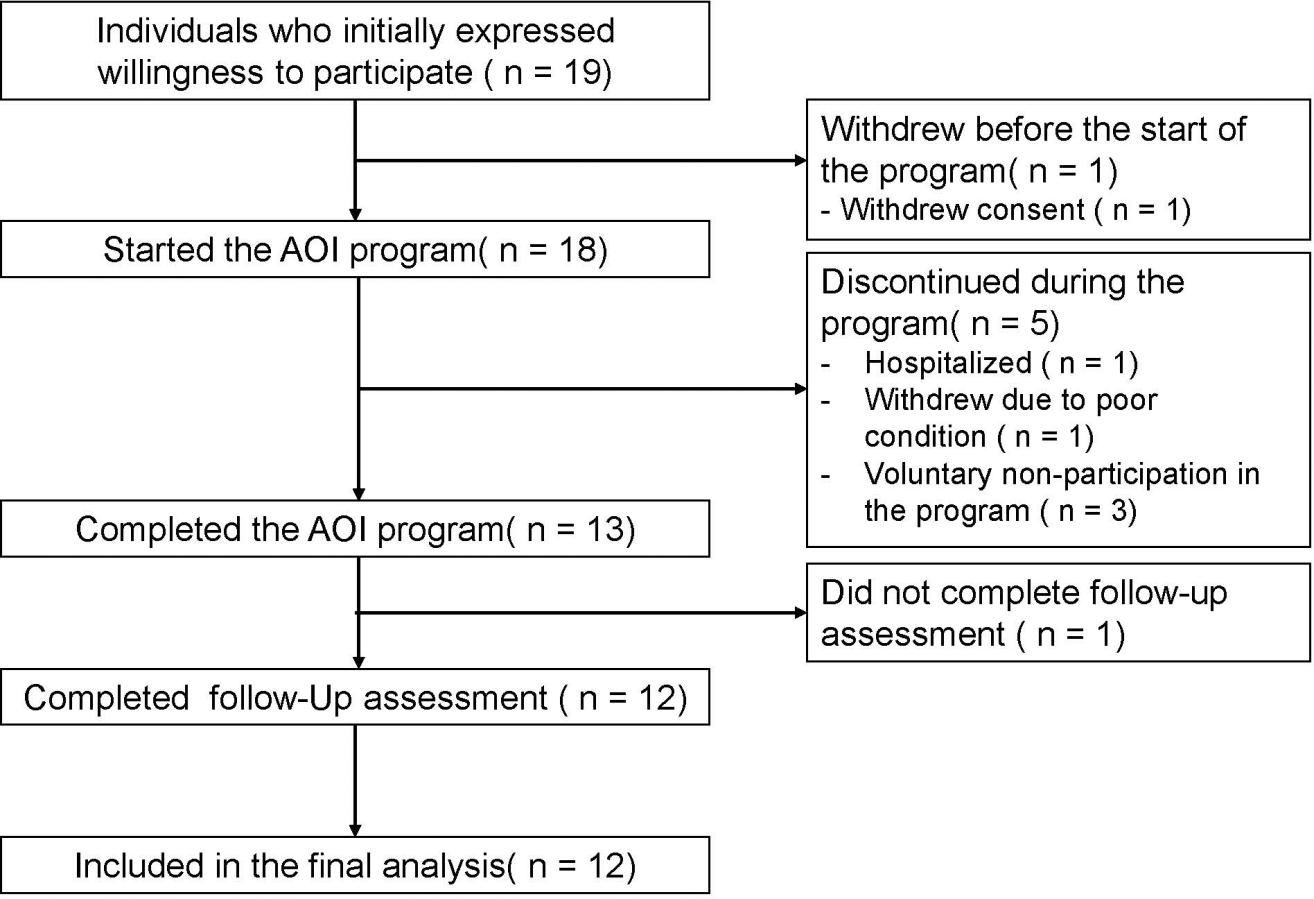
Flow of participants through the single-arm pilot feasibility intervention study.

Participants were recruited from a total of four facilities in Japan, and 19 individuals initially expressed willingness to participate in the study. Of them, one withdrew consent before the start of the program, one was hospitalized, one withdrew due to poor physical condition, three dropped out because of voluntary non-participation in the program, and one dropped out from the follow-up assessment. The flow of participants through the single-arm pilot feasibility intervention study is shown in **Figure 1**. As a result, 12 participants were included in the study. Participant characteristics are shown in **Table 1**. The participants showed variability in several clinical and functional characteristics, including the total duration of daycare use and baseline social functioning. This heterogeneity should be considered when interpreting the changes in outcome measures.

**Table 1:** Demographic information (n=12).

Age (y, mean ± SD)	55.8 ± 6.44
Sex	
Male	6
Female	6
Other	0
Household composition (Duplicate)	
Lived with others	
With family	4
With non-family (ex: friends/partner)	1
With group home member	2
Alone	5
Primary source(s) of income (multiple responses allowed)	
Disability pension	12
Public assistance	2
Part-time work	1
Education	
Junior high school graduate level	5
High school graduate level	4
University graduate level	3
Diagnosis	
Schizophrenia	11
Bipolar disorder	1
Age at first illness onset (y, mean ± SD)	25.0 ± 8.72
Total duration of daycare use (y, mean ± SD)	14.6 ± 6.26
Total duration of hospitalization (y, mean ± SD)	5.06 ± 5.34
Service use (number of participants)	
Daycare service only	4
Use of additional services in combination (multiple responses allowed)	8
Community service center	2
Home-visit nursing service	4
Employment support service	2
Home helper service	3

#### 3.1.2 Changes in outcome measures across intervention and follow-up

The scores for each measure are presented in **Table 2**. Comparisons across baseline, post-intervention, and follow-up using the Friedman test showed no significant changes in the primary or secondary outcomes. BPRS was the only measure to show a significant overall time effect. However, *post-hoc* pairwise comparisons did not reach significance, although there was a trend towards improvement from baseline to post-intervention (p = 0.089) and from baseline to follow-up (p = 0.12). In an exploratory descriptive observation, participants with baseline BPRS scores ≥30, indicating moderate to severe symptom levels, appeared to show lower community participation on the TUCP-J.

**Table 2:** Outcomes at pre-, post-, and follow-up assessments.

Outcome measure	Pre, median (Q1–Q3)	Post, median (Q1–Q3)	Follow up, median (Q1–Q3)	χ^2^	df	Effect size (Kendall’s W)	*p*
RAS	75.5 (73-87.2)	72 (66.2-81.2)	79 (76-83.8)	2.65	2	0.111	.266
TUCP-J							
Participation amount	36 (26.2-55.0)	23.5 (14.7-50.0)	16.5 (7.75-37.0)	7.30	2	0.30	.02*
Number of important activity areas	14.0 (4.00-21.2)	14.5 (5.00-25.2)	16.5 (3.75-26.2)	1.43	2	0.059	.49
Breadth of participation	4.50 (3.75-7.25)	3.50 (3.00-4.50)	4.00 (2.75-5.00)	4.79	2	0.199	.09
Breadth ratio	0.52 (0.33-0.81)	0.46 (0.22-0.67)	0.31 (0.13-0.72)	3.8	2	0.15	.15
Participation sufficiency	0.57 (0.48-0.77)	0.53 (0.17-0.67)	0.37 (0.19-0.76)	2.51	2	0.10	.28
SFS	88 (71.5-126.5)	94 (75-122)	91 (75-119)	3.70	2	0.15	.157
GAF	68 (60-72)	69 (61-79)	69 (62.2-79.2)	0.737	2	0.030	.692
BPRS	27.0 (21.5-35.0)	21.5 (19.5-24.2)	22.5 (19.5-24.5)	8.67	2	0.36	.013*

Values are presented as medians (Q1–Q3). Changes over time were analyzed using the Friedman test. Effect sizes are reported as Kendall’s W. *p < .05; **p < .01. Post hoc pairwise comparisons with the Bonferroni correction were performed for outcomes with significant overall differences, but no significant pairwise differences were found after correction.

### 3.2 Interviews with occupational therapists

#### 3.2.1 Participants’ characteristics

Semi-structured interviews were conducted with four occupational therapists who played central roles in implementing AOI. The interviews primarily explored the kinds of skills they used as occupational therapists when delivering AOI.

#### 3.2.2 Analysis of the interviews

The interviews were transcribed verbatim and analyzed using content analysis. A total of 133 codes were generated and organized into two overarching themes. The first theme comprised three categories and 24 subcategories related to competencies used in AOI delivery (**Table 3**). The second theme comprised three categories and 18 subcategories related to contextual and cultural adaptations and implementation barriers in Japanese psychiatric daycare settings (**Table 4**).

**Table 3:** Occupational therapists’ competencies used to deliver group-based AOI in Japanese psychiatric daycare settings.

Category	Subcategory	Illustrative narratives
A1. Therapeutic relational stance	Acceptance of the client’s verbal and non-verbal expressions	*“I continued to listen to the patient’s repetitive narratives while grappling with clinical uncertainty regarding the most appropriate response…”* *“While perceiving the patients’ resistance toward inquiries into their daily lives, the I maintained accepting presence.”*
	Maintenance of a non-judgmental stance	*“The intervention was conducted with an emphasis on empathic understanding of the individual, rather than pointing out the rightness or wrongness of actions.”*
	Rapport and equality	*“I consciously set aside the authoritative stance of “treating the patient” to foster a more equal partnership.”*
	Provision of supportive encouragement	*“The fundamental approach was to provide support that allows individuals to gain their own insights.”* *“I encouraged participation with the hope that “perhaps they can take the next step.”*
	Avoiding over-solicitation	*“I reflected on whether my invitations might have felt like a form of pressure or accusation.”*
	Recovery-oriented perspective	*“I ensured that the patients had the agency to determine their own priorities within the intervention.”* *“The program was initiated based on the specific difficulties and needs expressed by the participants.”* *“I deepened my understanding of the person’s life and relationships through our conversations.”*
A2. Group process management and implementation adjustment	Maximizing comprehensibility	*“I used a whiteboard to write down and share information visually with the group.”*
	Directing, monitoring, and fostering group-based interaction	*“I demonstrated a direction that encouraged all members to engage in collective discussion.”* *“I encouraged participants to read their worksheets aloud to the group.”* *“I maintained a supportive presence to allow participants to engage in peer-to-peer dialogue.”* *“I intervened to ensure the group was a positive experience, helping members feel that “it was good to talk” and “I am not alone.”*
	Inclusive group management	*“I provided follow-up support to ensure all participants could keep pace with the group.”* *“I repeated the process of completing one section of the worksheet at a time followed by group sharing to maintain a unified pace.”* *“I ensured that everyone had finished writing before moving on to the sharing phase.”* *“I provided one-on-one side-by-side support for participants who struggled with concentration or fell behind in the process.”*
	Maintaining psychological safety	*“I recognized that specific skills are required to manage and steer the group effectively.”*
	Group process management	*“I emphasized assessment during the initial phase of the program and adjusted my response as the sessions progressed.”*
	Assessing individual capacity	*“It was necessary to discern whether each participant was capable of speaking at that moment.”*
A3. Scaffolding participation and task engagement	Expanding possibilities	*“I identified various activities from the patients’ narratives and worked to expand them into different areas of their lives.”*
	Clarifying focal points	*“I had participants underline specific parts related to the topics discussed in the group or areas that were easy to imagine concretely to help them focus.”*
	Role distribution based on strengths	*“I assigned specific roles to participants based on their individual strengths.”*
	Anticipating outcomes	*“Since many participants felt a sense of achievement even with the continuation of short-term goals, I anticipated that they would feel “it was good I did it,” and I felt comfortable with that direction.”*
	Modifying program content	*“I incorporated mood checks and games, and made worksheet tasks optional when participants appeared fatigued.”*
	Grading	*“I provided “grading” (step-by-step adjustment) for goals that were initially too large for the participant.”*
	Strategic proposing	*“I found it necessary to make specific suggestions for those who were unable to choose anything on their own.”*
	Concretization of steps	*“I engaged in deeper discussions to examine and plan concrete steps.”*
	Selecting appropriate materials	*“I selected materials and tools that were best suited for the specific needs of the group.”*
	Integration with broader programs	*“I developed activities that evolved from the initial program into other therapeutic interventions.”*
	Enhancing motivation	*“I encouraged participants to focus on what they could do and the merits of their engagement.”*
	Individualized communication	*“I spoke to participants individually to emphasize how specific aspects of the program would be a ‘plus’ for their personal situation.”*

**Table 4:** Contextual and cultural adaptations and implementation challenges in delivering group-based AOI in Japan.

Category	Subcategory	Illustrative narratives
B1. Adaptations to reduce interpersonal exposure	Ensuring the right to remain silent	*“I guaranteed the right not to speak, acknowledging that some participants felt resistance toward discussing personal matters.”*
	Ensuring safe feedback	*“I ensured that corrective feedback or “pointing out” was conducted individually to maintain psychological safety.”*
	Difficulty in providing corrective feedback	*“I found it difficult to provide corrective feedback on the activities they usually perform. I couldn’t bring myself to say, ‘Not that way, but…’ When trying to take a step further from an abstract level, it often simplified back into their usual routine.”*
	Avoiding over-solicitation (A1)	*“I reflected on whether my invitations might have felt like a form of pressure or accusation.”*
	Rapport and equity (A1)	*“I consciously set aside the authoritative stance of “treating the patient” to foster a more equal partnership.”*
B2. Adaptations to enhance accessibility and discussability	Selecting group-appropriate topics	*“Since the group happened to consist of men of a similar age, topics such as employment and sexual orientation emerged. It was meaningful that the group’s cohesion allowed for the expression of how they spent their time.”*
	Identifying only overt issues	*“I limited my interventions to pointing out issues only when the number of selected activities was clearly excessive.”*
	Incorporating oral reading	*“I had the participants take turns reading the worksheets aloud to the group.”*
	Preparation to avoid confusion	*“I avoided the task of taking out worksheets during the session to prevent confusion and maintain focus.”*
	Clarifying focal points (A3)	*“I had participants underline specific parts related to the topics discussed in the group or areas that were easy to imagine concretely to help them focus.”*
	Maximizing comprehensibility (A2)	*“I used a whiteboard to write down and share information visually with the group.”*
	Concretization of steps (A3)	*“I engaged in deeper discussions to examine and plan concrete steps.”*
B3. Implementation barriers	Insufficient communication of the purpose	*“It was difficult for the patients to visualize participating in a new intervention, and some found it hard to accept.”* *“I felt that either it was difficult to imagine or the purpose was not fully communicated. The intervention felt so different from the usual programs that it created a sense of incongruity, making it difficult to convey the intended meaning.”*
	Difficulty in providing corrective feedback	*“I found it difficult to provide corrective feedback on the activities they usually perform. I couldn’t bring myself to say, ‘Not that way, but…’ When trying to take a step further from an abstract level, it often simplified back into their usual routine.”*
	Anxiety regarding risks	*“There was a struggle within the occupational therapist between the risk of relapse and the desire for change through new activities. I felt that simply increasing the frequency of what they usually do would be safe, but changing the framework itself carried inherent risks.”*
	Limitations of evaluation methods	*“I felt that if there were more objective evaluation tools available, we would be able to capture changes more effectively.”*
	Discrepancy between participants and program content	*“It is difficult to recruit individuals who seem unlikely to change. Yet, change is exactly what is needed. It might be better to gain a deeper understanding of each person’s characteristics and tailor the program more closely to them.”*
	Predominance of verbal communication	*“I felt that experiential components are necessary. Relying solely on verbal communication limits the number of people who can participate.”* *“It would be easier to implement if there were elements focused on physical functions (e.g., exercise).”*

##### 3.2.2.1 Occupational therapists’ competencies used in delivering group-based AOI

First, the findings identified the competencies used by occupational therapists during the 3-month group-based AOI implemented in this study. Specifically, three major categories were identified: Therapeutic relational stance; Group Process Management and Implementation Adjustment; and Scaffolding participation and task engagement.

Therapeutic relational stance included the fundamental interpersonal attitudes and perspectives that occupational therapists adopted when providing intervention. Specifically, it included receptive forms of engagement such as Acceptance of the client’s verbal and non-verbal Expressions and Maintenance of a non-judgmental Stance. In addition, the recovery perspective, which constitutes the foundational framework of AOI, was also reflected in the interviews, as illustrated by statements such as: “I ensured that the patients had the agency to determine their own priorities within the intervention.” and “The program was initiated based on the specific difficulties and needs expressed by the participants.”

Next, Group Process Management and Implementation Adjustment is described. This category included competencies that enabled participants to engage in the program safely and effectively, as well as strategies that made use of group dynamics. For example, participants’ understanding of the program was enhanced through strategies such as, “I used a whiteboard to write down and share information visually with the group.” In addition, occupational therapists assessed each participant’s abilities (Assessing individual capacity) and worked to create an inclusive structure, as reflected in the statement: “I provided one-on-one side-by-side support for participants who struggled with concentration or fell behind in the process.”

Finally, the category of Scaffolding participation and task engagement is described. This category captured the occupational therapists’ skills required to support and refine the tasks included in the program. Representative examples included basic techniques such as Grading and Enhancing Motivation, as well as Modifying program content and Individualized communication. Furthermore, the skill of Integration with broader programs, which is consistent with the central AOI concept of expanding activity, was also identified.

#### 3.2.3 Contextual and cultural adaptations and implementation challenges in delivering group-based AOI in Japan

Next, the findings related to Contextual and Cultural Adaptations and Implementation in delivering the Japanese group-based AOI are presented. Specifically, three major themes were identified: Adaptations to reduce interpersonal exposure; Adaptations to enhance accessibility and discussability; and Implementation barriers.

First, Adaptations to reduce interpersonal exposure suggested that adjustments were made to address participants’ resistance toward the self and self-expression within the group setting. Specifically, it was reported that Ensuring the right to remain silent was guaranteed in response to these concerns, and that feedback was also delivered in ways characterized as Ensuring safe feedback. Furthermore, in the context of Japanese daycare, Difficulty in providing corrective feedback was reported: “I found it difficult to provide corrective feedback on the activities they usually perform. I couldn’t bring myself to say, ‘Not that way, but…’ When trying to take a step further from an abstract level, it often simplified back into their usual routine.” In relation to this, occupational therapists were also found to engage in Avoiding over-solicitation, to restrain corrective comments (Identifying only overt issues), and to emphasize Rapport and equity.

Next, Adaptations to enhance accessibility and discussability showed that the situation was adapted so that a greater number of participants could engage more comfortably, through strategies such as Selecting group-appropriate topics (“Since the group happened to consist of men of a similar age, topics such as employment and sexual orientation emerged. It was meaningful that the group’s cohesion allowed for the expression of how they spent their time.”) and Preparation to avoid confusion. In addition, the use of oral reading helped ensure access to and participation in the group, and Maximizing comprehensibility and Concretization of steps were also utilized for this purpose.

Finally, several concrete Implementation barriers were identified. In terms of communication, Insufficient communication of the purpose and Difficulty in providing corrective feedback were reported. Difficulties related to Predominance of verbal communication were also described (“I felt that experiential components are necessary. Relying solely on verbal communication limits the number of people who can participate”). Furthermore, as described in the following statement, Anxiety Regarding Risks was also reported: “There was a struggle within the occupational therapist between the risk of relapse and the desire for change through new activities. I felt that simply increasing the frequency of what they usually do would be safe, but changing the framework itself carried inherent risks.” It was also reported that Discrepancy between participants and program content underlay these concerns: “It is difficult to recruit individuals who seem unlikely to change. Yet, change is exactly what is needed. It might be better to gain a deeper understanding of each person’s characteristics and tailor the program more closely to them.” This concern should not be interpreted as indicating that recovery-oriented interventions are appropriate only for individuals who appear ready or likely to change. Rather, it highlights the need to adapt the intervention to participants whose recovery processes may be diverse, gradual, and not immediately visible, while maintaining the recovery-oriented assumption that change remains possible in different forms and at different paces.

## 4 Discussion

In this pilot study, no significant effects were observed in the key exploratory outcome of personal recovery (RAS) or the other exploratory outcomes. However, this finding should be interpreted in light of the very small sample size. As this was a pilot feasibility study, the sample size was not determined based on a formal power calculation for detecting intervention effects. Therefore, the absence of statistically significant changes should not be interpreted as evidence of no effect, because the study was likely underpowered and at risk of Type II error. Rather, the quantitative findings should be regarded as exploratory and used to inform the design of a future adequately powered RCT, including sample size estimation, outcome selection, and consideration of variability in outcome responses.

In this mixed-methods pilot feasibility study, integration was not intended to confirm intervention effectiveness through convergence between quantitative and qualitative findings. Rather, the quantitative component was used to describe exploratory changes in recovery, participation, social functioning, and symptoms, while the qualitative component was used to contextualize these changes by examining therapists’ experiences of implementation, cultural and institutional adaptation, and perceived barriers. Therefore, the integration of findings aimed to identify the conditions under which group-based AOI may be more feasible and responsive in Japanese psychiatric daycare settings.

### 4.1 Integration of quantitative and qualitative findings

The integration of quantitative and qualitative findings suggests that the absence of clear intervention effects should not be understood simply as a lack of potential effectiveness of AOI in Japan. Rather, the combined findings point to several implementation conditions that may need to be optimized before measurable changes in recovery and participation can be expected. These conditions can be understood in terms of intervention dose, fit between participants and the program, and readiness for activity change.

First, because AOI involves the reconstruction of everyday life and the formation of new habits, sufficient intervention and follow-up periods are essential to detect change. Although this study was conducted within a realistic framework as a pilot trial, the intervention dose may have been insufficient to detect measurable effects. Previous research has also suggested that a 3-month intervention period may be inadequate (9), and evidence from other psychosocial interventions (4, 11) similarly suggests that the appropriate duration of implementation remains an open question.

Second, the findings related to TUCP-J should be interpreted cautiously. Although the decline in TUCP-J participation and the descriptive observation that participants with higher baseline BPRS scores appeared to show lower community participation may suggest a possible relationship between symptom burden and participation, this relationship was exploratory and cannot be interpreted causally. The observed decline in participation may have reflected several overlapping factors. For some participants, the reflective and goal-setting components of AOI may have increased awareness of the gap between desired and actual participation, leading to lower self-rated participation. This interpretation is consistent with previous findings showing that subjective evaluations among people with schizophrenia can be influenced by insight, self-reflection, depressive symptoms, and discrepancies between subjective and objective assessments (25, 26). In addition, the intervention itself may have placed a certain burden on participants by asking them to reflect on everyday routines, consider activity change, and engage in group-based discussion. This burden may have been particularly salient for participants with higher symptom severity, verbal difficulties, anxiety about interpersonal exposure, or limited readiness for change (27).

It is also possible that the group-based format did not sufficiently fit the needs of some participants. In psychiatric daycare settings in Japan, participants may be accustomed to structured group activities, but not necessarily to individualized reflection, explicit goal setting, or verbal sharing of personal experiences within a group. Therefore, the decrease in TUCP-J participation may reflect not only symptom severity, but also a possible mismatch between the intervention demands and participants’ readiness, communication style, or current stage of recovery. Additional support may therefore be required, including visualization, demonstration, non-verbal activities, and one-to-one bridging. Future studies should examine more carefully which participants are most likely to benefit from AOI, what level of support is needed, and how group-based delivery can be adapted to reduce intervention burden while maintaining the core components of AOI. While the BPRS scores showed an overall significant improvement over time, the differences between specific time points did not reach significance in *post-hoc* comparisons, suggesting a preliminary trend. This change may reflect the combined influence of this intervention, including AOI elements that promote the structuring of daily life, as well as psychoeducational components. However, the present study design does not allow causal attribution of symptom change, and future studies including control groups are needed.

Participant heterogeneity should also be considered when interpreting the quantitative findings. The participants varied in the duration of daycare use and baseline social functioning, suggesting differences in recovery stage, functional status, and readiness for activity change. At the same time, most participants had used psychiatric daycare services for many years, indicating that daycare may have functioned as a central place for daily routine, social contact, and community participation. This heterogeneity may have contributed to variability in outcome responses and made it difficult to detect consistent changes across the small sample.

Taken together, the absence of clear intervention effects in this study may have been related not only to insufficient intervention dose, but also to the fitness between participants and the program, the forms of support required to sustain participation, and the contextual conditions associated with group-based delivery. To understand more concretely what these preconditions involve, the qualitative findings derived from interviews with occupational therapists are important. By clarifying the competencies required to deliver AOI in a group format, the contextual and cultural adaptations needed to make participation possible, and the barriers encountered in implementation, it becomes possible to interpret the issues of dose, fitness, and readiness suggested by the quantitative findings at a more practical level. Importantly, the qualitative findings should not be understood merely as a *post-hoc* explanation for non-significant quantitative results. Rather, they identify practical and contextual mechanisms that may have shaped participants’ engagement with AOI and that should be addressed in future adaptation and trial design.

### 4.2 Implications for AOI implementation and adaptation suggested by the qualitative findings

Several competencies identified in **Table 3**, particularly those categorized under relational and ethical stance and scaffolding participation and task engagement, appear consistent with the core principles of AOI, including collaborative engagement, non-judgmental support, and the gradual facilitation of meaningful occupational change (8).

In contrast, the competencies categorized as group process management and implementation adjustment suggest an important extension beyond the core competencies typically emphasized in AOI. When AOI was delivered in a group format, OTRs were required not only to support individual reflection and choice-making, but also to manage the collective process of participation, pacing, mutual interaction, and psychological safety. Although AOI has been implemented in group formats (10), there has been little prior discussion in the literature regarding the competencies, clinical judgments, and underlying principles required for its delivery in such settings. The present study therefore makes a practical contribution by providing concrete insights into the competencies, clinical judgments, and guiding principles involved in group-based AOI delivery.

These group-management competencies became particularly salient in the Japanese psychiatric daycare context, where participants’ hesitancy toward self-disclosure, sensitivity to interpersonal evaluation, and difficulty engaging with unfamiliar intervention formats appeared to require further contextual adjustments, as illustrated in **Table 4**. A notable adaptation concerned the way corrective input was delivered. Rather than providing explicit corrective feedback in the group setting, occupational therapists described the need to individualize and soften feedback to preserve psychological safety. This suggests that, in this context, the effectiveness of AOI depends not only on facilitating reflection, but on doing so in ways that minimize interpersonal exposure and perceived criticism.

In interpreting these findings, culture should not be treated as a fixed national characteristic. Rather, cultural adaptation is understood here as the process of adjusting an intervention to fit the interaction between broader cultural values, communication norms, service structures, and the everyday relational contexts in which the intervention is delivered. Therefore, the findings should not be read as indicating that silence, hesitancy toward self-disclosure, or sensitivity to interpersonal evaluation are uniquely Japanese traits. Instead, they may reflect how general principles of group-based and recovery-oriented practice require different degrees of emphasis and different forms of operationalization in a specific cultural and service context.

The emphasis on protecting the right to remain silent indicates that participation in group-based AOI could not be equated with verbal self-disclosure. In this setting, allowing participants to engage without immediate pressure to speak appeared essential for maintaining safety and readiness. It should be noted that protecting psychological safety, allowing silence, and building rapport are not culture-specific practices in themselves; they are also central principles of recovery-oriented and group-based interventions more broadly. However, the present findings suggest that, in this context, these principles required particular emphasis and careful operationalization. This finding suggests that cultural adaptation may involve redefining participation itself, from active verbal contribution to graded and self-paced engagement. This interpretation should be understood cautiously, as the present study did not directly compare Japanese and Western contexts. Nevertheless, previous cross-cultural research has suggested that Japanese and East Asian individuals may be more cautious in disclosing private or sensitive information than Western participants (15, 28) and that lower relational mobility is associated with lower self-disclosure (29). Psychiatric daycare settings in Japan may represent a relatively low-relational-mobility environment, where participants often remain in long-standing and difficult-to-change relationships within the same service setting. In such contexts, verbal self-disclosure and corrective feedback in a group may carry greater interpersonal risk. At the same time, long-standing group relationships may also facilitate rapport, familiarity, and a sense of safety, thereby creating conditions in which self-disclosure becomes easier over time. Thus, the key implementation issue is how to shape a group environment that reduces interpersonal risk while also using existing relationships to support gradual trust, safety, and readiness for self-expression.

Therefore, these findings should not be interpreted as indicating that hesitancy toward self-disclosure or sensitivity to interpersonal evaluation are uniquely Japanese phenomena. Rather, they may partly reflect the interaction between broader cultural communication patterns, including collectivist orientations, and the long-standing predominance of group-based programs in Japanese psychiatric daycare and occupational therapy practice. In this sense, the findings highlight the need to consider both cultural and service-contextual factors when implementing AOI within this setting. The selection of group-appropriate topics suggests that adaptation was required not only at the level of therapist behavior, but also at the level of content. Topics appeared more discussable when they resonated with shared life circumstances within the group, indicating that relevance and discussability were socially, rather than purely individually, constituted.

Another key adaptation involved the use of visual and oral supports, such as whiteboards, oral reading, and clarification of focal points. These strategies appeared to reduce cognitive and communicative demands and made the program more accessible in a group setting. This suggests that the implementation of AOI in this context required not only linguistic translation, but also interactional and instructional translation.

The findings further suggest that a central adaptation challenge lay in balancing the transformative intent of AOI with participants’ and therapists’ need for safety. Because AOI invites participants to reconsider habitual routines and try new forms of engagement, its implementation in this setting required careful titration of novelty. Change needed to be introduced gradually enough to remain tolerable, meaningful, and non-threatening.

Taken together, the contextual and cultural adaptations identified in **Table 4** suggest that implementing AOI in Japanese psychiatric daycare required the intervention to be made less exposing, less abstract, and less abrupt, while preserving its core aim of supporting occupational change.

Bernal et al. (30) proposed a framework for cultural adaptation in mental health interventions, identifying dimensions such as Language, Persons, Metaphors, Content, Concepts, Goals, Methods, and Context. The present findings particularly suggest challenges related to Methods and Context within this model. More specifically, the results indicate a need to adjust AOI to fit the clinical structure of Japanese psychiatric daycare, where group-based programming is common, despite AOI’s core emphasis on individualized goal setting and reflection. In addition, the findings may also point to the relevance of Goals and Concepts, insofar as the meaning of recovery itself may differ across cultural contexts. This interpretation is consistent with prior work suggesting that recovery may be understood differently across cultures, and that, in collectivist societies, it may be more strongly linked to the restoration of social roles and family relationships (31, 32). It is also in line with findings from Japanese participants emphasizing the importance of social roles in recovery. It also resonates with qualitative findings from Japan indicating that personal recovery is shaped through relationships with others, places where individuals can feel accepted and connected, and experiences of stigma (6). In the present AOI implementation, this relational understanding of recovery was reflected in group processes such as peer-to-peer dialogue, role distribution based on participants’ strengths, rapport-building, and the selection of group-appropriate topics. Thus, social roles in this context refer not only to broader roles such as worker, family member, or community member, but also to more immediate roles within the intervention group, including being a peer, listener, speaker, or supportive group member. This suggests that adapting AOI may require attention not only to individual goal setting, but also to how group activities create opportunities for participants to experience meaningful roles in relation to others.

Furthermore, previous literature has suggested that collectivist orientations in Asian contexts may themselves be used as therapeutic resources in recovery-oriented practice (33). From this perspective, the present findings suggest that future adaptation of AOI may need to consider not only how recovery is defined, but also how it is facilitated. Research remains limited regarding how collectivism, harmony, and socially embedded role expectations in East Asian contexts shape recovery processes, and even fewer studies have examined how such factors should inform intervention design. The present findings therefore should not be read as evidence that silence, rapport-building, or safe feedback are uniquely Japanese practices, but rather as indicating that these generally important group-process principles may require different degrees of emphasis and different forms of operationalization depending on cultural and service contexts. Therefore, the present study suggests the need for further development of AOI in ways that retain its theoretical foundations while making it more responsive to East Asian clinical and cultural contexts.

This study is significant as the first pilot study to implement AOI across multiple psychiatric daycare settings in East Asia, while also identifying concrete clinical and cultural adaptation challenges in the implementation process. In particular, the findings illuminate the interaction between existing group-centered practice structures and the more individualized orientation of AOI. These insights provide an important foundation for future cultural adaptation and intervention design, and they may contribute to the development of an AOI model that remains faithful to its theoretical framework while being better suited to East Asian clinical contexts.

### 4.3 Limitations

Several limitations should be acknowledged. First, the quantitative component was limited by the single-arm design, small sample size, and absence of randomization. Because no control group was included, changes observed over time cannot be causally attributed to AOI and may have been influenced by confounding factors, such as usual daycare participation, concurrent services, medication changes, symptom fluctuation, or other contextual factors. Therefore, any improvement in BPRS should be interpreted as an exploratory trend rather than evidence of an intervention effect. In addition, because this was a pilot feasibility study, the sample size was not based on a formal power calculation. The quantitative analyses were therefore underpowered, and the absence of statistically significant changes may reflect Type II error rather than a true lack of intervention effect. The inclusion of multiple outcome measures further increased the difficulty of interpreting changes across outcomes. Accordingly, the quantitative findings should be considered exploratory and used primarily to inform outcome selection, sample size estimation, and the design of future controlled and preferably randomized trials.

Second, participant and group-level heterogeneity may have influenced the findings. Participants varied in duration of daycare use, baseline social functioning, symptom severity, and recovery stage. Although this heterogeneity reflects the clinical reality of psychiatric daycare settings in Japan, it may have affected participants’ readiness to engage with AOI, their responses to the intervention, and the variability of outcome changes. In addition, some AOI groups included both research participants and non-participants. Differences in motivation, engagement, and participation in assessments may therefore have influenced group dynamics and intervention outcomes. Future studies should consider stratification or adjustment for key baseline characteristics, such as duration of daycare use, social functioning, symptom severity, and recovery stage, and should examine how group composition affects implementation and outcomes.

Third, intervention fidelity was not fully evaluated. The introduction of IMR psychoeducation in Section 3 meant that part of the intervention structure differed from the original AOI. Although this modification was intended to improve contextual fit, and selected IMR materials were incorporated only within the information provision component to support discussion of activity, health, and participation, the intervention did not constitute a strict fidelity-tested delivery of the original AOI manual. In addition, because the AOI workbook does not include a formal fidelity framework or checklist, fidelity was considered conceptually in relation to the core principles of AOI rather than assessed using a standardized checklist. Future research should clarify the core components of AOI, establish fidelity indicators, distinguish core elements from adapted components, and further develop culturally adapted psychoeducational materials that are more directly aligned with AOI.

Fourth, outcome measurement was limited. Although the Japanese versions of the RAS and TUCP-J have established reliability and validity, the present study relied heavily on self-report measures to assess recovery and participation. These measures may not have been sufficiently sensitive to short-term behavioral changes in daily activity patterns or community participation during the intervention and follow-up period. In addition, self-reported recovery and participation may be influenced by cultural factors, including how participants interpret personal change, express needs, and evaluate their own participation in relation to others. Future studies should incorporate behavioral and observational indicators, such as activity records, attendance or participation logs, clinician-rated assessments, and qualitative accounts of everyday activity change, alongside self-report measures.

Fifth, the qualitative component also has limitations. The occupational therapist interviews were not originally designed to investigate cultural factors, and interpretations of cultural influences therefore remain exploratory. In addition, interviews were conducted with only four occupational therapists, which limits the breadth and transferability of the qualitative findings. Researcher bias may also have influenced data collection and interpretation, as members of the research team were involved in both AOI implementation and qualitative analysis. Selection bias is also possible, as participating therapists may have had a particular interest in AOI, recovery-oriented practice, or cultural adaptation. Furthermore, the study did not include service users’ voices; therefore, the findings primarily reflect therapists’ perspectives rather than participants’ lived experiences of engagement, burden, self-disclosure, or perceived benefit. Future studies should include larger and more diverse samples of therapists, service users, and other stakeholders to triangulate perspectives and strengthen the transferability of the findings.

Finally, the follow-up period was short and did not permit evaluation of sustained changes in everyday life behaviors. Given that AOI aims to support changes in activity patterns and habit formation, behavioral and participation-related changes may require a longer period to emerge and stabilize. Future studies should include longer-term follow-up assessments, such as 6 months or longer, to examine sustained and delayed effects.

## Data Availability

The original contributions presented in the study are included in the article/supplementary material. Further inquiries can be directed to the corresponding author.
